# Evaluation of Electric Field Integral Voltage Measurement Method of Transmission Line Based on Error Transmission and Uncertainty Analysis

**DOI:** 10.3390/s21134340

**Published:** 2021-06-25

**Authors:** Jiarui Fan, Cheng Ai, Aofei Guo, Xiaojun Yan, Jingang Wang

**Affiliations:** State Key Laboratory of Power Transmission Equipment and System Security, Chongqing University, Chongqing 400044, China; 20183560@cqu.edu.cn (J.F.); 20183372@cqu.edu.cn (C.A.); 20183362@cqu.edu.cn (A.G.); yxjdmu@cqu.edu.cn (X.Y.)

**Keywords:** transmission line voltage measurement, numerical integration of electric field, error transmission, uncertainty evaluation

## Abstract

Electric field numerical integration algorithms can realize the non-contact measurement of transmission line voltage effectively. Although there are many electric field numerical integration algorithms, lack of a comprehensive comparison of accuracy and stability among various algorithms results in difficulties in evaluating the measurement results of various algorithms. Therefore, this paper presents the G-L (Gauss–Legendre) algorithm, the I-G-L (improved Gauss–Legendre) algorithm, and the I-G-C (improved Gauss–Chebyshev) algorithm and proposes a unified error propagation model of the derived algorithms to assess the accuracy of each integration method by considering multiple error sources. Moreover, evaluation criteria for the uncertainty of transmission line voltage measurement are proposed to analyze the stability and reliability of these algorithms. A simulation model and experiment platform were then constructed to conduct error propagation and uncertainty analyses. The results show that the G-L algorithm had the highest accuracy and stability in the scheme with five integral nodes, for which the simulation error was 0.603% and the relative uncertainty was 2.130%. The I-G-L algorithm was more applicable due to the smaller number of integral nodes required, yet the algorithm was less stable in achieving the same accuracy as the G-L algorithm. In addition, the I-G-C algorithm was relatively less accurate and stable in voltage measurement.

## 1. Introduction

Traditional methods for contact voltage measurements for transmission lines are limited in their application due to their large size, high price, and narrow frequency domain response [1,2,3]. These methods have gradually become unsatisfactory for the requirements of an extensive network in a transmission and distribution system. On the other hand, non-contact methods for voltage measurement have become a popular research direction because of their advantages of convenient operation and high safety [4,5,6,7]. Moreover, they allow accurate measurement of voltage for the transmission lines. Regardless of applying non-contact optical sensors or non-contact electric field coupling sensors [8,9,10,11,12], the transmission line voltage can be obtained by inverse calculation with the exact measurement data of the electric field. The commonly used methods for inverse solution are the field-source inverse calculation problem and field-source numerical integration.

The field-source inverse calculation problem is challenging to promote for large-scale usage due to the complexity of calculating the capacitance matrix and solving field-source equations [13,14,15]. Therefore, the numerical integration method is optimal for non-contact measurement of transmission line voltage. Wang et al. proposed inverse calculation of the transmission line voltage using a D-dot sensor and Gauss numerical integration in 2018, and their experimental test results were reliable, with a relative error size of below 0.5% [16,17]. However, this measurement was carried out under relatively ideal conditions. Furthermore, the research group derived different integral algorithms from the Gauss numerical integral to calculate the transmission line voltage, including G-L (Gauss–Legendre) numerical integration, I-G-L (improved Gauss–Legendre) numerical integration, G-C (Gauss–Chebyshev) numerical integration, and G-K (Gauss–Kronrod) numerical integration, where the highest measurement accuracy was obtained (up to 0.3%) with G-K [18,19,20,21]. In addition, the researchers presented a scheme for reconstructing the parameters of the integral nodes to improve the accuracy of the solution results [22]. Although most publications related to field-source numerical integration problems have proposed many numerical integration methods and optimal schemes for reducing the measurement error, there is a lack of an effective comparison and evaluation concerning the accuracy and stability of various electric field integration algorithms. The literature mentioned above [17,18,19,20,21] selected different integration algorithms to compare the accuracy of the final measurement results. The current research does not discuss the possible error sources in actual applications and the influence of error propagation on the final voltage measurement results, resulting in the inability to optimize the numerical integration method for calculating the transmission line voltage from the sources of error, and the difficulties in practical application. In addition, these studies have not discussed evaluation of the uncertainty of the field-source numerical integration methods thoroughly. The lack of specific quantification and measurements of the practical use of the electric field numerical integration methods will lead to the inability to measure the stability and reliability of the results in actual voltage measurements, which prevents the effective monitoring of voltage in transmission and distribution systems.

Stratakis et al. analyzed the uncertainty in electromagnetic field strength measurements, but the researchers did not discuss the application of numerical integration methods to voltage measurement [23,24,25]. Some researchers [26,27,28] only studied the respective measurement performance of sensors but did not precisely determine and express the uncertainty of electric field measurement. Yan et al. proposed an uncertainty assessment method for voltage measurement, yet the established uncertainty was not precise due to the lack of an analysis of error sources with the voltage sensor [29].

This paper proposes a unified error transfer model and uncertainty evaluation standard that can be applied to all the electric field numerical integration methods to improve the relevant discussion. Moreover, the authors realized a comparison of accuracy and stability among different integration algorithms to provide theoretical support for applying the electric field numerical integration methods.

**Figure 1:**
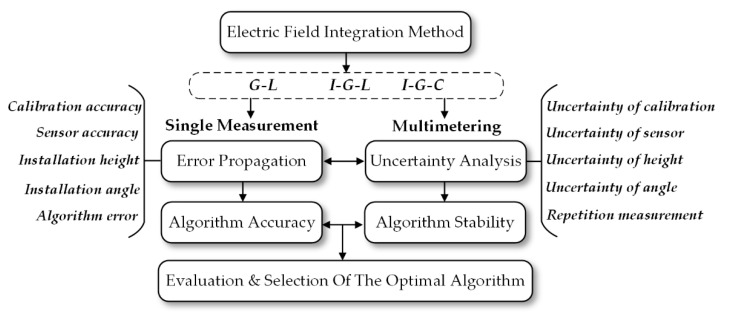
Article framework.

This paper is organized as shown in Figure 1. The first section introduces the principle of Gaussian numerical integration and three electric field numerical integration methods derived from Gaussian numerical integration. The second section discusses various sources of error in actual transmission line voltage measurements and establishes the error transfer model. On this basis, the third section puts forward an evaluation standard for the uncertainty of voltage measurement. Finally, in the fourth part, the magnitude of error propagation (error transfer) and the uncertainty of the methods mentioned above were calculated to evaluate their accuracy and stability by simulation and experiments.

## 2. Principles of Numerical Integration of Electric Fields and Typical Algorithms

### 2.1. Calculation of the Electric Field under the Transmission Line

For power frequency transmission lines, it can be assumed that the charges on the lines are line charges. Supposing there are *m* parallel and infinite transmission wires, the relationship between the voltage of the wires and their respective equivalent line charges can be expressed as [30]:

(1) $$\left[\begin{array}{c} \begin{array}{c} U_{1}\\ U_{2} \end{array}\\ \begin{array}{c} \cdots \\ U_{m} \end{array} \end{array}\right]=\left[\begin{array}{cccc} \lambda_{11} & \lambda_{12} & \cdots & \lambda_{1m}\\ \lambda_{21} & \lambda_{22} & \cdots & \lambda_{2m}\\ \cdots & \cdots & \cdots & \cdots \\ \lambda_{m1} & \lambda_{m2} & \cdots & \lambda_{mm} \end{array}\right]\left[\begin{array}{c} Q_{1}\\ \begin{array}{c} Q_{2}\\ \begin{array}{c} \cdots \\ Q_{m} \end{array} \end{array} \end{array}\right]$$

where *U* is the voltage matrix of each wire, *Q* is the equivalent charge matrix on each wire, and λ is the *m*-order square matrix composed of the potential coefficients of each wire. In the plane of right-angled coordinates perpendicular to the transmission line, the horizontal and vertical components of the electric field’s strength at any point *P* (*x, y*) can be expressed as:

(2) $$\left\{ \begin{array}{l} E_{x}=\frac{1}{2\pi \varepsilon_{0}}\sum_{i=1}^{m}Q_{i}\left(\frac{x-x_{i}}{d_{ip}^{2}}-\frac{x+x_{i}}{D_{ip}^{2}}\right)\\ E_{y}=\frac{1}{2\pi \varepsilon_{0}}\sum_{i=1}^{m}Q_{i}\left(\frac{y-y_{i}}{d_{ip}^{2}}-\frac{y+y_{i}}{D_{ip}^{2}}\right) \end{array}\right.$$

where *x_i_* and *y_i_* represent the position of the wire, in which *i* is the sequence number; *d_ip_* is the distance between the wire and point *P*; *D_ip_* is the distance between the image charge of the wire and point *P*; and *m* is the number of wires.

From Equations (1) and (2), the relationship between the electric field strength under the transmission line and the voltage of the transmission line cannot be expressed by the elementary function formula, so it is difficult to use the Newton–Leibniz formula to calculate the line voltage directly. Therefore, it is better to use a numerical integration algorithm to perform the electric field inverse calculation.

### 2.2. Numerical Integration of the Electric Field and Gaussian Derivative Algorithms

**Figure 2:**
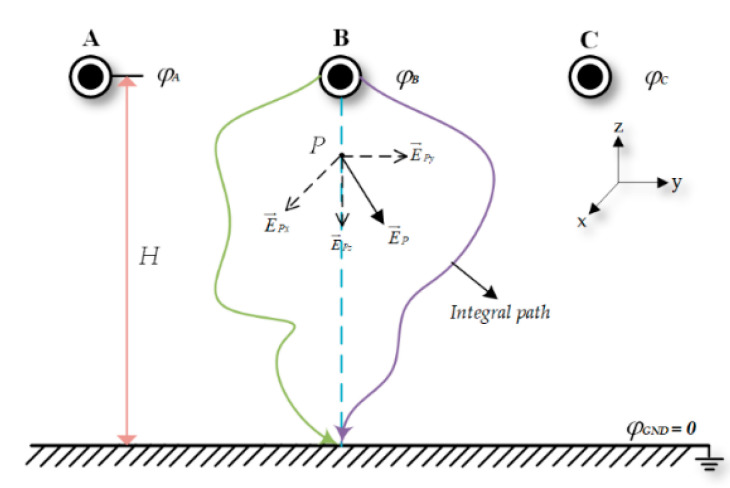
Electric field integral method for measuring transmission line voltage.

In Figure 2, setting the ground as the zero-potential point, the integration path can be chosen casually, from the beginning of the transmission line surface to the ground. The spatial electric field vector can then be integrated along the integration path to obtain the voltage of the transmission line, $\varphi_{l}$.

(3) $$\underset{Integral\;path}{∭}\vec{E}(x,y,z)d\vec{x}d\vec{y}d\vec{z}=\varphi_{l}$$

The spatial electric field intensity vector can be orthogonally decomposed into the electric field components in three directions; the vertical path between the transmission wire and the ground is selected as the integration path, and the transmission line voltage can be obtained by the integral of the one-dimensional vertical component *Ez.*

(4) $$\varphi_{l}=\int_{0}^{H}E_{z}dz$$

By using Gaussian numerical integration instead of continuous integration, the line voltage $\varphi_{l}$ can be uniformly expressed as:

(5) $$\varphi_{l}=\int_{0}^{H}E_{z}dz\approx \sum_{j=1}^{n}A_{j}{E(z}_{j})$$

where *A_j_* is the integration coefficient and *E*(*z_j_*) is the electric field intensity along the vertical direction at the discrete integration node *z_j_* on the integration path. The other Gaussian derivative algorithms in this section are all based on the Gaussian prototype integration algorithm mentioned above. The difference lies in the selection of different integration nodes and quadrature coefficients.

#### 2.2.1. Gauss–Legendre Integral Method

The G-L method is an improved algorithm of Gauss prototype integration. Its weight function is 1 and *H* is the height of the transmission line to the ground. The normalized formula is:

(6) $$z=\frac{H}{2}+\frac{H}{2}\cdot t$$

We convert the integral interval of *z* ϵ (0, *H*) to the interval of *t* ϵ (−1, 1) to obtain the Gauss–Legendre electric field quadrature formula:

(7) $$\varphi_{l}=\frac{H}{2}\cdot \int_{-1}^{1}E(t)\cdot dt=\frac{H}{2}\cdot \sum_{j=0}^{n}C_{j}E\left(\frac{H}{2}+\frac{H}{2}\cdot t_{j}\right)$$

The position of the normalized integration node *t_j_* is the zero point of the Legendre polynomial, which is a series of fixed values. The Legendre polynomial and the quadrature coefficient are:

(8) $$P_{n+1}(t)=\frac{1}{2^{n}n!}\frac{d^{n}}{dt^{n}}|{\left(t^{2}-1\right)}^{n}|$$

(9) $$C_{j}=\frac{2}{(1-t_{j}^{2})[{\left(P_{n+1}^{\prime }(t_{j})\right]}^{2}}$$

#### 2.2.2. Gauss–Chebyshev Integral Method

Unlike G-L, the weight function of the Gauss–Chebyshev integration method is $1/\sqrt{{1-x}^{2}}$, and the electric field integration interval is also normalized to (−1, 1). The Gauss–Chebyshev numerical integration formula can be expressed as:

(10) $$\varphi_{l}=\sum_{j=0}^{n}\frac{H}{2}\cdot \frac{\pi }{n+1}E\left(\frac{H}{2}+\frac{H}{2}\cdot \cos\frac{2j+1}{2(n+1)}\pi \right)$$

#### 2.2.3. Improved Piecewise Integration Method

**Figure 3:**
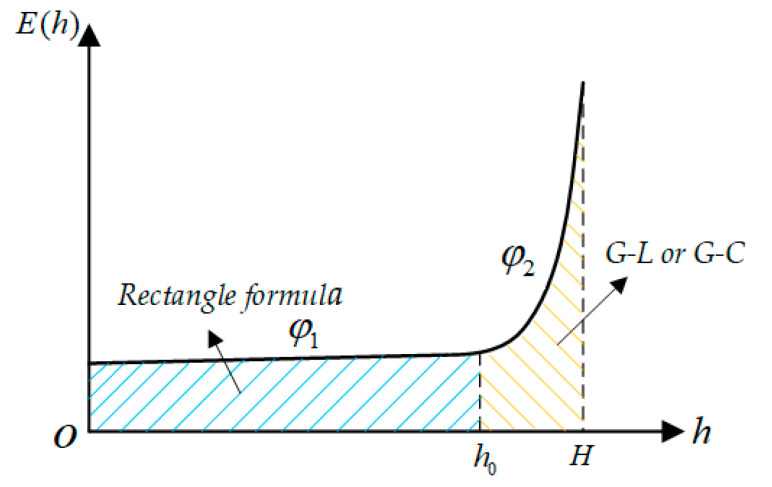
Voltage calculation by the piecewise integral method.

Due to the distribution characteristics of the spatial electric field, a segmented improvement algorithm can be used to reduce the number of integration nodes while ensuring integration accuracy. Figure 3 shows the distribution of the electric field in the space below the transmission lines, which can be divided into a sudden change area and a non-mutation change area.

In the non-mutation region (0, *h*_0_), the electric field changes smoothly and the number of integration nodes in the interval is small. The rectangular formula can be used to approximate the voltage $\varphi_{1}$ in this interval:

(11) $$\varphi_{1}=\int_{0}^{h_{0}}E(x)\cdot dx=h_{0}\cdot E\left(\frac{h_{0}}{2}\right)$$

In the sudden change area of the electric field (*h*_0_, *H*), normalization can be performed first and then the G-L or G-C algorithm can be used to solve the voltage $\varphi_{2}$ in this interval.

Finally, the voltage of the transmission line of the improved piecewise integral method is the sum of the voltages in the two intervals:$\;\varphi_{l}=\varphi_{1}+\varphi_{2}$.

#### 2.2.4. Truncation Error of the Numerical Integration Method

By using numerical integration of finite discrete nodes instead of continuous Riemann integration, the result will inevitably have a particular error. This type of error in numerical integration is called truncation error in mathematics. The size of the truncation error is closely related to the method of numerical integration and algebraic precision.

Here are the truncation error expressions of the three integration algorithms mentioned above. The truncation error expression of the G-L algorithm is:

(12) $$R_{L}=\frac{2^{2n+3}{\left[(n+1)!\right]}^{4}}{(2n+3){\left[(2n+2)!\right]}^{3}}E^{\left(2n+2\right)}(\eta ),\;\eta \in (-1,1)$$

The truncation error expression of the G-C algorithm is:

(13) $$R_{C}=\frac{2\pi }{2^{2n}(2n)!}E^{\left(2n\right)}(\eta ),\;\eta \in (-1,1)$$

The I-G-L algorithm uses both the trapezoidal formula and the G-L algorithm. The truncation error expression of the trapezoidal formula is:

(14) $$R_{T}=-\frac{E^{\prime \prime }(\eta )}{12}{\left(b-a\right)}^{3},\;\eta \in (a,b)$$

Since the expression of electric field intensity is very complicated and the size of the truncation error is related to the number of integration nodes selected, it is complicated and impractical to use mathematical formulas to find the truncation errors of the three integration algorithms, so we considered the method of simulation to calculate the truncation error of the integral node under each node and the specific content method. The specific content method will be elaborated in Section 3.3 of the article.

In addition to the three integration algorithms mentioned above, there are many other electric field numerical integration methods. Voltage measurement results vary from the different integration algorithms, yet a reliability evaluation among the different algorithms is lacking at present.

Therefore, an error propagation model of the electric field numerical integration method for measuring the voltage of the transmission line is proposed to judge the accuracy of various methods, and the uncertainty evaluation criteria are established, based on the error, to measure the stability and reliability of the algorithm.

## 3. Error Propagation Analysis

Analyzing the error sources in the practical application of electric field numerical integration can offer suggestions for optimizing the practical application of the integration algorithms and comparing the accuracy of the results of different kinds of integration method at the same time. According to the principles and actual situation of the electric field integration method, the primary sources of measurement error are calibration error, sensor accuracy error, installation angle error, installation height error, and the algorithm error of the numerical integration itself.

### 3.1. Calibration Error and Accuracy Error

It is necessary to calibrate the non-contact electric field sensor in advance before using it. Because the calibration instrument has a specific precision limit, it will produce a calibration error. The maximum measurement error of the calibration instrument is regarded as the calibration error $\Delta_{1}$, which is given at the time of production.

At the same time, the sensor itself has an inevitable accuracy error. For the accuracy of the non-contact sensor, the maximum relative error η% is generally used to measure its accuracy. Therefore, the absolute error $\Delta_{2}$ of electric field measurement accuracy can be expressed as: $\Delta_{2}{=E}_{m}\;\times \;\eta \%$, where *E_m_* is the measured value of the electric field.

### 3.2. Installation Error

**Figure 4:**
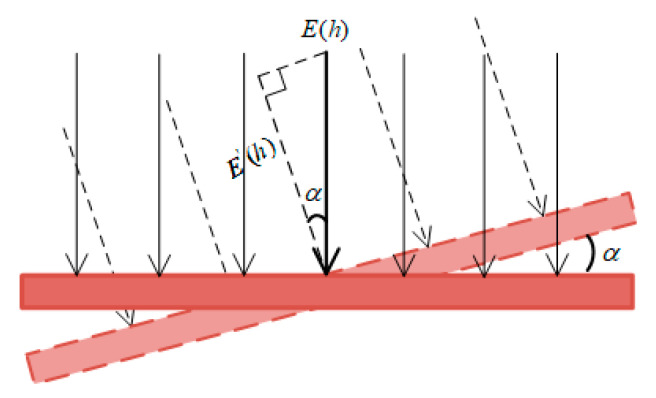
Schematic diagram of angle installation error.

Installation error is divided into angle installation error and height installation error, which belong to the system errors. The non-contact sensor has certain requirements for the placement angle of the sensor, because it should measure the projection of spatial electric fields in the vertical direction of the wire. When there is a fixed deviation between the actual installation angle and the ideal installation angle, the angle installation error $\Delta_{3}$ of the electric field will be produced. A schematic diagram of the installation angle offset α of the electric field sensor is shown in Figure 4.

Since the measurement error $\Delta_{3}$ produced by the sensor installation angle and the angle deviation are not purely trigonometric functions in actual situations, the angle sensitivity coefficient *k*_1_ is introduced here to reflect the degree of the influence of the installation angle error on the magnitude of the electric field error. Finally, the expression of the angle error $\Delta_{3}$ of the electric field can be obtained as:

(15) $$\Delta_{3}=k_{1}\times (1-\cos\alpha )\times E_{m}$$

For the electric field numerical integration method, the quadrature coefficient *A_i_* corresponds to the height position of the measuring point. Once the placement height of the sensor deviates from the theoretical height, the measured electric field intensity modulus will not be the electric field value at the specified height position in the numerical integration algorithm, which will result in a height error.

**Figure 5:**
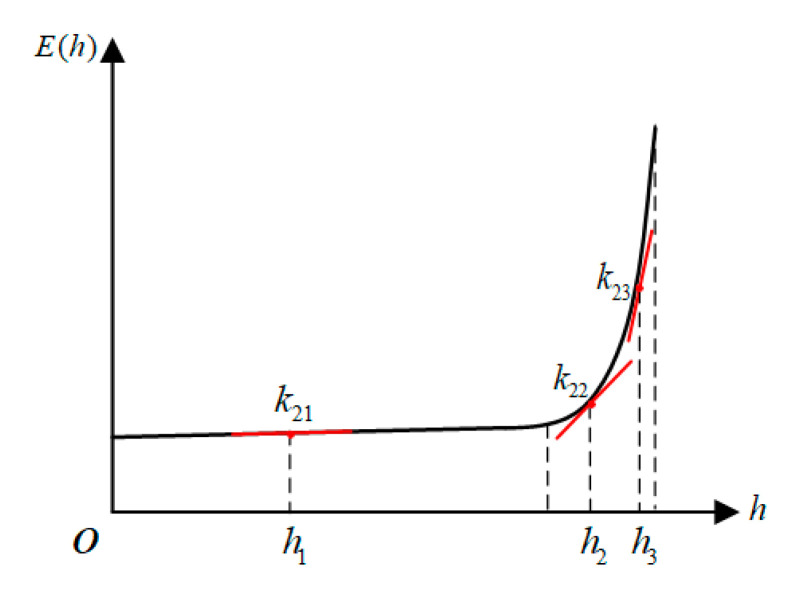
Sensitivity of electric field strength to height.

Due to the distribution characteristics of the electric field in space, the electric field intensity at different heights has a different sensitivity to height. Thus, the height sensitivity coefficient *k*_2_ is introduced here to reflect the influence of the installation height offset on the height installation error of the electric field $\Delta_{4}$. The size of *k*_2_ is related to the installation height, which is shown in Figure 5. When a height error of Δ*h* occurs, the electric field error $\Delta_{4}$ can be expressed as:

(16) $$\Delta_{4}=k_{2}\times \Delta h\times E_{m}$$

### 3.3. Numerical Integration Algorithm Error

Section 2.2.4 presented an overview of the truncation error for the numerical integration algorithm. The error size which is calculated by simulation method will be explained carefully in this section. Through a simulation experiment of transmission line voltage measurement, the electric field intensity at each integration node position can be measured. The electric field integration algorithm was then used to solve the simulation calculation value $\varphi_{sc}$ of the transmission line voltage. Compared with the voltage excitation value $\varphi_{s}$ added in the simulation, the expression of the numerical integration algorithm error $\Delta_{5}$ was obtained as:$\;\Delta_{5}=\varphi_{s}-\varphi_{sc}$.

### 3.4. Error Propagation

Regarding the error size of the electric field intensity on a single integrating node, the sources of electric field error mentioned earlier are independent of each other, and the final synthesized electric field measurement error of a single node can be obtained as:

(17) $$\varepsilon_{n}=\Delta_{n1}+\Delta_{n2}+\Delta_{n3}+\Delta_{n4}$$

where $\varepsilon_{n}$ represents the measurement error of the combined electric field intensity of the *n*^th^ integral node.

When the voltage of the transmission line is measured, the integration nodes are also independent of each other, so the measurement error transfer $\Delta_{\varphi l}$ of the final voltage can be expressed as:

(18) $$\Delta_{\varphi l}=\sum_{n=1}^{m}A_{n}\cdot \varepsilon_{n}+\Delta_{5}$$

where *m* indicates that, in total, *m* integration nodes have been selected, and $\Delta_{5}$ varies with the selection of the integration method.

## 4. Uncertainty Assessment

In order to realize effective monitoring of the voltage of a transmission and distribution system, the reliability of the measurement results needed further evaluation based on error analyses of the three electric field integration algorithms. Thus, it was necessary to establish uncertainty evaluation criteria determined by Type A and B uncertainty. The standard uncertainty of each electric field scheme was then obtained and used to obtain the extended uncertainty of the measurement results for transmission line voltage.

### 4.1. Standard Uncertainty of the Electric Field

Equation (5) shows that the uncertainty of the measurement results of transmission line voltage are only determined by the uncertainty of the electric field measurement results. Furthermore, the unified error propagation model shows that uncertainty is generated by four sources of error in electric field measurement. When the electric field magnitude of the same integration node is repeatedly measured under the same conditions, the standard uncertainty magnitude of the electric field can be obtained by synthesizing the following five components.

#### 4.1.1. Calibration and Accuracy Uncertainty

The uncertainty introduced by the accuracy error of both the calibration devices and electric field sensors are defined as Type B uncertainty. The maximum possible error of the calibration device is taken as the half-width of its possible value interval. Since the uncertainty caused by the calibration error is random, the probability distribution is considered as the average distribution so that the calibration uncertainty component can be obtained as:$\;u_{1}\left(E\right)=\Delta_{1}/\sqrt{3}$.

The sensor accuracy uncertainty *u*_2_(*E*) is handled in a similar was to the calibration error uncertainty. With its maximum accuracy error as the half-width of the maximum interval’s possible values occurring, the accuracy standard uncertainty component is: $u_{2}\left(E\right)=\Delta_{2}/\sqrt{3}$.

#### 4.1.2. Angle and Height Uncertainty

The uncertainty *u*_3_(*E*) caused by the mounting angle error is still regarded as a Type B uncertainty component. The half-width of the interval of possible value fluctuations is represented as the maximum electric field measurement error $\Delta_{3}$. Considering that when placing the sensor, the experimental operator will place it according to the predetermined standard as much as possible, the probability of not generating the angular mounting error should be maximum. Thus, the probability distribution is considered as a triangular distribution with the inclusion factor $\;\sqrt{6}$. The uncertainty *u_3_*(*E*) can be expressed as: $u_{3}\left(E\right)=\Delta_{3}/\sqrt{6}$.

Similar to the angular standard uncertainty, the maximum measurement error in the electric field due to height error $\Delta_{4}$ is considered to be a triangular distribution with the inclusion factor $\sqrt{6}$. The height standard uncertainty *u*_4_(*E*) can be expressed as: $u_{4}\left(E\right)=\Delta_{4}/\sqrt{6}$.

#### 4.1.3. Standard Uncertainty of Repeated Measurement 

During the actual voltage measurement, repeated measurement uncertainties generated by accidental causes in multiple measurement processes are defined as Type A uncertainty, calculated by the Bessel formula. The standard uncertainty component *u_5_*(*E*) of repeated measurements is given as follows:

(19) $$\bar{E}=\frac{E_{1}+E_{2}+E_{3}+\ldots +E_{n}}{n}$$

(20) $$u_{5}(E)=\sqrt{\frac{\sum_{i=1}^{n}{\left(E_{i}-\bar{E}\right)}^{2}}{n(n-1)}}$$

#### 4.1.4. Standard Uncertainty of Electric Field 

The covariance coefficient between each component is zero and their impact on the electric field is independent, so the sensitivity coefficients of each component are 1. Thus, the electric field standard uncertainty is:

(21) $$u_{c}(E)=\sqrt{\sum_{i=1}^{5}u_{i}^{2}(E)}$$

### 4.2. Voltage Synthesis Uncertainty

From the principle of the numerical integration algorithm, it is known that the transmission line voltage is the value of the electric field at the integration node multiplied by the summation of the product coefficient. Because integration nodes are unrelated to each other, the magnitude of the partial derivative of the transmission line voltage on the electric field is the sensitivity coefficient of the uncertainty component. The sensitivity coefficient *C_i_* of the electric field at each integration node is:

(22) $$C_{i}=\frac{\partial \varphi_{l}}{\partial E_{i}}=A_{i}$$

Thus the synthetic uncertainty of the voltage measurement result *u_c_*($\varphi_{l}$) is:

(23) $$u_{c}(\varphi_{l})=\sqrt{\sum_{i=1}^{m}A_{i}^{2}u_{c}{\left(E\right)}_{i}}$$

where *u_c_*(*E_i_*) represents the standard uncertainty of the electric field measurement result at the *i*^th^ integration node and *m* represents the total number of integration nodes.

Finally, taking the inclusion factor $k_{\varphi }=2$, the extended uncertainty *U* of the transmission line voltage measurement result can be obtained as $U=k_{\varphi }\cdot u_{c}\left(\varphi_{l}\right)$.

## 5. Evaluation of Voltage Measurement Methods

### 5.1. Electric Field Distribution under the Transmission Line

**Figure 6:**
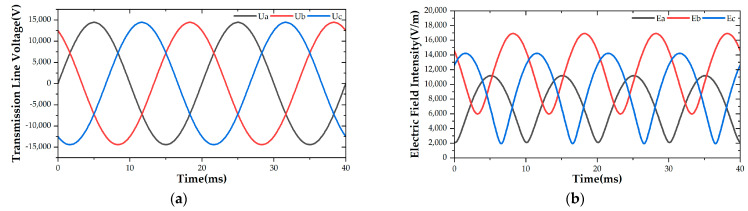
(**a**) Three-phase transmission line voltage; (**b**) electric field intensity below the transmission line.

A transient simulation model of a three-phase horizontally distributed transmission line was constructed with Ansoft Maxwell, in which the transmission line height was 1.5 m and the phase space was 0.6 m. Initially, the spatial electric field distribution under the transmission line was simulated. The results of variations in the transmission line voltage and electric field at 1.25 m high measuring points are shown in Figure 6, indicating the that magnitude of the electric field under the transmission line changed periodically with the periodical change of the line voltage.

**Figure 7:**
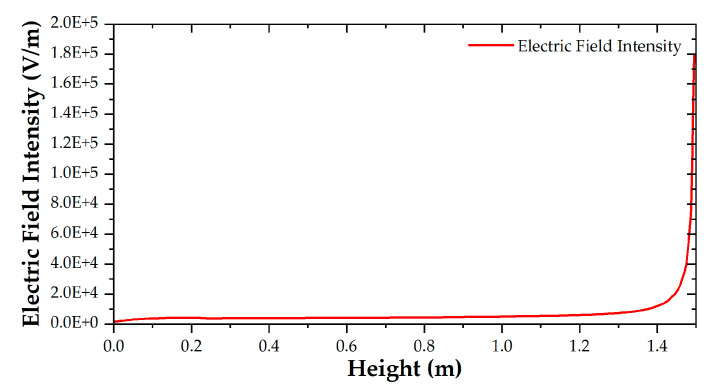
Distribution of the electric field under a B-phase transmission line.

The variation curve of the spatial electric field’s magnitude with the height of the B-phase transmission line at the initial time is shown in Figure 7. According to the electric field intensity distribution, the electric field under the transmission line can be divided into a non-mutation region and a sudden change region, which satisfies the conditions for using the segmental integration algorithm.

### 5.2. Error Simulation of the Electric Field Integral Voltage Measurement Methods

**Figure 8:**
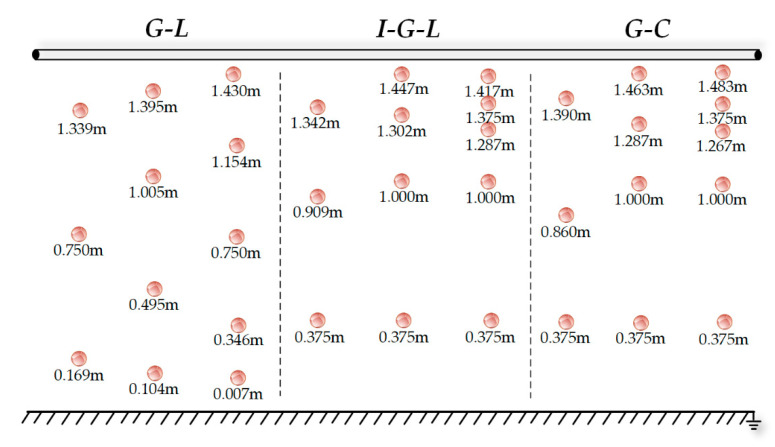
Position of integral nodes.

Next, a voltage measurement simulation was carried out. This section only discusses B-phase transmission line voltage measurement, since the voltage of each phase of the transmission line only has phase variation, and the A- and C-phase line voltage can be obtained using the same method. The G-L, I-G-L, and I-G-C algorithms were used for electric field measurements, and the error analyses concerning different algorithms and different integral nodes schemes on voltage measurement were conducted to evaluate the accuracy of the algorithms. The positions of the integral nodes of different schemes are shown in Figure 8.

**Figure 9:**
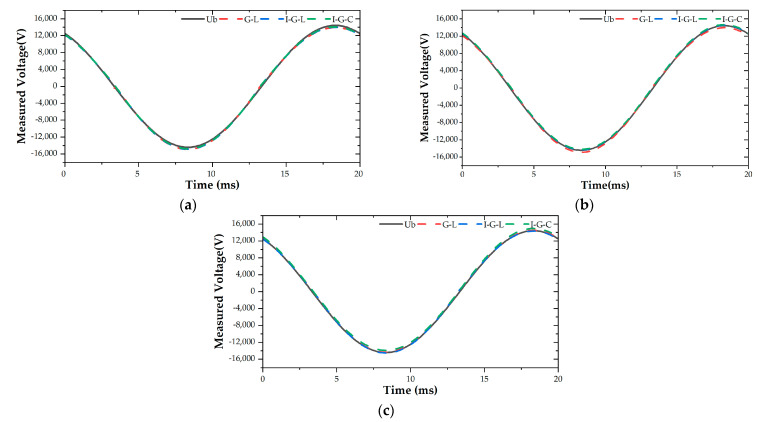
Voltage measurement results based on different algorithms: (**a**) three integral nodes; (**b**) four integral nodes; (**c**) five integral nodes.

According to the formulae in Section 2.2, the B-phase transmission line voltage under different schemes at each measurement moment of a cycle was calculated. The plotted results are shown in Figure 9. The calculated measured voltage values follow the theoretical voltage values well in the figure, illustrating the feasibility of using the electric field numerical integration algorithm to measure transmission line voltage.

Next, an error analysis was carried out by selecting the moments corresponding to the peak of the theoretical voltage values and calculating the measurement errors of different schemes at the selected moments. The results of the measurement errors are shown in Table 1.

After analyzing the results of measurement errors, we found that different integration algorithms and different numbers of integration nodes had different effects on the voltage measurement error. The measurement error of the G-L algorithm was only 0.603% with a sufficient number of integration nodes (*m* = 5); the measurement error of the I-G-L algorithm was slightly larger than that of the G-L integration method by 0.15% when *m* = 5. Moreover, the error of the I-G-L algorithm was only 0.226% greater when *m* = 4 compared with that of G-L algorithm when *m* = 5; the measurement error of the I-G-C integration method was greater than 1.5%. The measurement error analysis indicated that the G-L algorithm had the highest accuracy with a sufficient number of integration nodes, the I-G-L algorithm had great practical value for its good accuracy and because it reduced the number of sensors effectively, and the I-G-C integration method had the relatively lowest accuracy.

### 5.3. Evaluation of Actual Measurement Uncertainty

#### 5.3.1. Experimental Platform Construction

To evaluate the uncertainty of the integration algorithms, an experimental platform of 10 kV three-phase horizontal transmission lines with a transmission height of 1.5 m was constructed. Multiple sets of data were collected to compare and evaluate the uncertainty of different algorithms.

**Figure 10:**
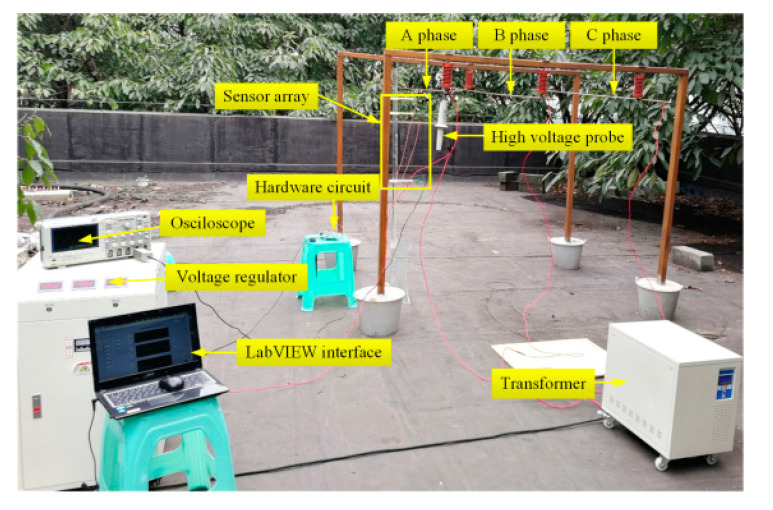
Actual experimental site.

The transmission line voltage measurement experiment site is shown in Figure 10. Insulators were installed under the wooden supporters, copper wires were hung between the insulators to simulate the three-phase transmission lines, and the high voltage probe was hung from the conductor to ensure its safety-grounding. The experimental equipment included a three-phase frequency transformer with a rated capacity of 20 kVA and a rated ratio of 500, and a three-phase frequency regulator with a rated capacity of 10 kVA, an input voltage of 380 V, and an output voltage of 0–400 V. Copper wires simulated the three-phase transmission lines, the three-phase power frequency voltage regulator regulated the voltage of the transmission line, and the oscilloscope measured the voltage value of the copper wires. The voltage measurement by different algorithms when *m* = 4 was conducted, and the D-dot sensors used to measure the electric field intensity were installed at the corresponding integral node positions shown in Figure 8.

#### 5.3.2. Uncertainty Evaluation and Analysis

Ten independent experiments were carried out repeatedly, and the uncertainty of each electric field algorithm was obtained, as shown in Table 2.

The maximum accuracy error of the experimental sensor was 1.5% and the calibration error was 0.5%; the uncertainty of accuracy and the relative uncertainty of calibration determined from this were 0.866% and 0.285%, respectively. According to the previous data, the angle error was set to no more than 0.5%, and the relative uncertainty was uniformly set to be 0.204%. The uncertainty of height, degree, and repeated measurements were obtained through experiments. Next, the relative uncertainty of voltage measurement was synthesized. The experiment results demonstrated that the G-L algorithm had the highest stability with a relative uncertainty of only 2.13%, followed by the I-G-L algorithm, and the I-G-C algorithm had the worst stability. Generally speaking, these algorithms are highly stable and suitable for practical measurement requirements because the relative uncertainty of the three algorithms is below 5%.

## 6. Conclusions

In this paper, the closely connected error transfer model and the uncertainty model were established for the electric field numerical integration algorithm. Furthermore, the accuracy and stability of transmission line voltage measurements applying the G-L algorithm, the I-G-L algorithm, and the I-G-C algorithm were comprehensively analyzed through simulations and experiments. According to the experimental and simulation results, we consider the I-G-L algorithm to be the most suitable for practical engineering applications because it can use fewer integration nodes while maintaining the accuracy and stability of the measurement results.

This paper proposes a comprehensive method for evaluating the reliability of the electric field integration algorithm by error propagation analysis and uncertainty analysis. The error propagation analysis focuses on evaluating the accuracy of a single result, and the uncertainty analysis focuses on the stability of multiple long-term results of the test algorithm. This method facilitates the evaluation of the accuracy and stability of practical steady-state and transient voltage measurements.

In following work, further comparisons of reliability among different electric field integration algorithms under various voltage measurement environments and transmission line models are needed. Moreover, the error transfer model and the uncertainty model need to be optimized to enhance the applicability of the accuracy and stability analysis.

## Figures and Tables

**Table 1:** Measurement errors of different schemes.

Algorithm	Point	$\left|\Delta_{\varphi l}\right|$	$|\Delta_{\varphi l}|\%$
G-L	*m* = 3	473.201	3.282%
	*m* = 4	466.707	3.237%
	*m* = 5	86.994	0.603%
I-G-L	*m* = 3	440.373	10.305%
	*m* = 4	119.495	0.829%
	*m* = 5	109.184	0.757%
I-G-C	*m* = 3	341.284	2.367%
	*m* = 4	235.910	1.636%
	*m* = 5	514.592	3.569%

**Table 2:** Measurement uncertainties of different schemes.

Algorithm Type	Measuring Point	Correction	Accuracy	Angle	Height	Repeat	Combined	
		*u*_1_(*E*)%	*u*_2_(*E*)%	*u*_3_(*E*)%	*u*_4_(*E*)%	*u*_5_(*E*)%	*u_c_*(*E*)%	$u_{c}(\varphi_{l})\%$
G-L	1	0.285	0.866	0.204	0.021	1.211	1.530	2.130
	2	0.285	0.866	0.204	0.038	1.288	1.591	
	3	0.285	0.866	0.204	0.104	1.201	1.528	
	4	0.285	0.866	0.204	0.208	1.197	1.533	
I-G-L	1	0.285	0.866	0.204	0.031	1.212	1.531	3.443
	2	0.285	0.866	0.204	0.075	1.308	1.609	
	3	0.285	0.866	0.204	0.193	1.213	1.543	
	4	0.285	0.866	0.204	0.308	1.131	1.499	
I-G-C	1	0.285	0.866	0.204	0.031	1.313	1.612	4.548
	2	0.285	0.866	0.204	0.075	1.283	1.589	
	3	0.285	0.866	0.204	0.198	1.321	1.629	
	4	0.285	0.866	0.204	0.297	1.221	1.566	

## Data Availability

The datasets generated and analyzed during the current study are available from the corresponding author on reasonable request.
